# The impact of unfairness experience on cooperative behavior revealed by ultimatum game-public goods game integration: an ERPs study

**DOI:** 10.3389/fpsyg.2025.1602181

**Published:** 2025-09-01

**Authors:** Yu-Jie Wang, Yao-Zhong Liu

**Affiliations:** ^1^Mental Health Education and Counseling Center, Guangdong Industry Polytechnic University, Guangzhou, China; ^2^School of Management, Jinan University, Guangzhou, China

**Keywords:** inequity aversion, cooperation, fairness, social decision-making, event-related potentials

## Abstract

Fairness critically shapes cooperative behavior in social dilemmas, yet the neurocognitive mechanisms linking unfairness experiences to cooperation remain underexplored. Twenty-four participants (*M*_age_ = 19.50 years, *SD* = 1.06) completed the Ultimatum Game (UG) with three proposal types (fair, moderately unfair, and extremely unfair) while event-related potentials (ERPs) were recorded, followed by the Public Goods Game (PGG) to assess cooperation. Behavioral results revealed that participants exhibited robust inequity aversion, rejecting moderately and extremely unfair UG proposals at significantly higher rates than fair one. Exposure to unfairness reduced subsequent PGG contributions, underscoring fairness as a priority over material gains. ERPs results showed that unfair proposals elicited stronger medial frontal negativity (MFN), reflecting norm violation detection, while fair proposals evoked larger P300 amplitudes, indexing reward valuation. Exploratory analyses revealed that P300 amplitudes positively related to cooperative behavior, suggesting reward-related neural activity facilitates post-inequity cooperation. These findings elucidate behavioral patterns of inequity aversion in interactive games and their neurophysiological correlates, advancing the understanding of how fairness preferences regulate cooperative decision-making.

## 1 Introduction

Pursuit of justice and maintenance of fairness represent universal imperatives in human societies. Amid globalization and technological revolutions, unfairness has emerged as a critical determinant of human cooperative behavior. Individuals exhibit inequity aversion during interactive games, where unfair treatment evokes resentment and dissatisfaction, prompting costly punitive actions to restore fairness even at personal economic expense (Fehr and Gachter, 2002; Gwako et al., 2006). In resource allocation, people balance profit-maximizing motives (characteristic of the *homo economicus*) with concerns for others' welfare and procedural fairness. Unfairness exposure triggers a decision conflict wherein *inequity aversion* operates as a prosocial dilemma: although self-interest optimization may temporarily breach fairness norms, such violations paradoxically intensify neural responses associated with social disgust (Tanaka et al., 2024). This underscores inequity aversion as a pivotal predictor of cooperative behavior in strategic interactions. While behavioral experiments confirm that individuals exposed to fair distributions exhibit stronger cooperation than those subjected to unfairness (Kong et al., 2024), the neurocognitive mechanisms underlying inequity aversion and their predictive power for cooperative behavior remain underexplored. To address this gap, we employ ERPs to investigate neural dynamics during exposure to varying degrees of fairness in resource allocation and their association with subsequent cooperative decisions.

## 2 Literature review

### 2.1 Cooperative behavior in social dilemmas

Human cooperative behavior is essential for the rational exploitation of limited resources and the successful completion of team tasks. Individuals in groups frequently encounter mixed-motivation situations where personal interests conflict with collective interests, or short-term gains oppose long-term benefits. It is precisely these dilemmas of mixed motivations that expose people to various social dilemmas (Dawes and Messick, 2000; Hauser et al., 2019). Researchers define cooperative behavior as an individual's behavioral choice in social dilemmas that suppresses short-term self-interest to maximize collective benefits (Dou et al., 2018; Henrich and Muthukrishna, 2021). When confronting conflicting interests, the ability to eschew hostile competition in favor of cooperative coexistence holds profound significance for enhancing personal wellbeing and advancing human society (Declerck et al., 2013; Rand, 2016; Yin et al., 2025).

To empirically investigate cooperative behavior, researchers commonly employ the ecologically valid public goods game (PGG) paradigm, which simulates real-world dilemmas through a “give-game” mechanism. In this paradigm, *N* participants receive experimental currency units (ECUs) and independently decide how much to contribute to a public account. The total contributions are multiplied (simulating resource growth) and equally redistributed to all members. Crucially, while higher aggregate contributions lead to greater public account growth and increased individual payoffs, free-riders (those contributing minimally or nothing) can exploit the system by retaining more ECUs than high contributors while still receiving equal shares. If no participant contributes, the public good collapses entirely. Therefore, an individual's contribution level directly indexes their cooperation intensity in public goods dilemmas (Dou et al., 2018; Sun et al., 2024).

### 2.2 Unfair experiences and cooperative behavior

Extensive experimental research demonstrates that individuals exhibit fairness preferences even while pursuing self-interest (Choshen-Hillel and Yaniv, 2011; Tsoi and McAuliffe, 2020). Fehr and Schmidt (1999) integrated this social preference for fairness into behavioral economics by proposing the concept of inequity aversion—the tendency to avoid unequal resource distributions (whether advantageous or disadvantageous) and even incur personal costs to achieve equality (Han et al., 2018; Fehr and Schmidt, 1999). Researchers typically operationalize IA through the Ultimatum Game (UG), where participants acting as Responders reject unequal allocation proposals from Proposers. The rejection rate serves as a validated proxy for IA intensity, a methodology robustly supported by empirical studies (Corradi-Dell'Acqua et al., 2013; Hu and Mai, 2021; Wang et al., 2022). While existing evidence confirms that individuals act as both *homo economicus* (maximizing monetary gains) and *homo socialis* (sacrificing self-interest to punish norm violators) in resource allocation contexts (Fehr et al., 2008; Nishi and Christakis, 2015; Tanaka et al., 2024), the mechanisms through which inequity aversion influences cooperative behavior in interpersonal interactions remain underexplored.

Real-world social dilemmas are inherently embedded in contexts of objective inequality, where individuals' decision-making processes are inevitably shaped by prior experiences of subjective unfairness (Hauser et al., 2019). At the macro level, wealth disparity and resource inequality amplify social dilemmas—a pattern resonating with Confucius's admonition in *Analects* 16.1: “*The greatest concern lies not in scarcity but in unfair distribution*”—indicating that distributive injustice jeopardizes social stability more severely than absolute poverty. Empirical evidence corroborates this ancient wisdom: Oishi and Kesebir (2015) analyzed socioeconomic data from 34 nations and demonstrated that national economic 3growth often fails to enhance collective wellbeing when accompanied by escalating economic inequality, as the psychological costs of resource polarization frequently negate the benefits of material prosperity. At the micro level, exposure to unfair conditions—such as socioeconomic status asymmetry or income inequality—systematically erodes the sustainability of altruistic, prosocial, and cooperative behaviors while heightening unethical conduct (Bechtel et al., 2018; Cheng et al., 2021; Du et al., 2021; Jachimowicz et al., 2020). These findings collectively position unfairness experiences as critical inhibitors of cooperative behavior in interactive games.

### 2.3 Neural mechanisms of inequity aversion

Humans' social preference for inequity aversion is rooted in distinct neural substrates. Pioneering fMRI research by Sanfey et al. (2003) revealed that unfair proposals activate emotion-related anterior insula, cognition-control-associated dorsolateral prefrontal cortex (DLPFC), and conflict-resolution-linked anterior cingulate cortex (ACC). Crucially, the activation intensity of the anterior insula exhibits a significant positive correlation with rejection rates of unfair offers—a finding that underscores the role of emotional processing in social decision-making (Sanfey et al., 2003). Complementing these results, Tabibnia and Lieberman (2007) demonstrated that fair proposals engage reward-processing regions, including the ventromedial prefrontal cortex (vmPFC), amygdala, ventral striatum, and orbitofrontal cortex (OFC), indicating that equitable outcomes function as intrinsic “reward stimuli.” While neuroimaging studies have mapped fairness-related brain networks (Fliessbach et al., 2012; Kish-Gephart, 2017; Wang et al., 2022), ERPs provide unparalleled temporal resolution advantages for dissecting inequity aversion dynamics in iterative games. Recent ERP evidence highlights two key components: MFN and P300.

The MFN component, peaking at 250–350 ms post-stimulus over frontocentral regions (with generators localized to the ACC; Gehring and Willoughby, 2002), reflects rapid semi-automatic evaluation of fairness violations during mid-stage processing (Leng and Zhou, 2010). Empirical studies consistently demonstrate that MFN amplitudes are enhanced for negative outcomes Boksem et al., (2011) and stimuli violating social norms (Li et al., 2025; Wang et al., 2017; Wu et al., 2011). In UG, unfair proposals elicit stronger MFN than fair offers (Spape et al., 2019), with (Boksem et al. 2011) further demonstrating that MFN sensitivity to unfairness correlates with individual fairness concerns. These findings collectively establish MFN as a neural marker of inequity sensitivity, where norm-deviant unfair proposals amplify negative deflections.

In contrast, the later-emerging P300 component (300–600 ms post-stimulus, maximal over parieto-occipital regions) indexes advanced cognitive processes during outcome evaluation, including emotional significance appraisal and attentional resource allocation (Li et al., 2025; Mussel et al., 2022). P300 primarily indexes advanced cognitive processes during the late-stage evaluation of fairness outcomes (Li et al., 2025; Mussel et al., 2022). Empirical evidence indicates that P300 amplitude reflects the integration of emotional significance appraisal and attentional resource allocation during decision-making (Hu and Mai, 2021; Leng and Zhou, 2010). Crucially, its amplitude is modulated by both outcome valence and reward magnitude, with positive feedback and larger monetary rewards eliciting enhanced P300 positivity (Bellebaum et al., 2010; Kobza et al., 2011). For instance, Xu et al. (2020) employed a third-party punishment paradigm to investigate neural responses to donation proposals, demonstrating that fair allocations evoke larger significantly lager P300 amplitudes than unfair ones. Converging evidence from UG studies further shows that fair proposals—which align with social norms and yield higher personal gains—consistently elicit more pronounced P300 responses than inequitable offer (Mussel et al., 2022; Wu et al., 2011). Collectively, these findings position P300 as a neural signature of fairness valuation, integrating both normative adherence and utilitarian benefit computations.

### 2.4 The current study

Despite extensive evidence that humans balance self-interest with fairness enforcement in social dilemmas, critical gaps persist in understanding how neural dynamics of unfairness processing (e.g., MFN/P300) calibrate cooperative decisions following unfair experiences. The current study integrates the UG and PGG to address these questions through a neurobehavioral lens. We hypothesize that: (1) Individuals will demonstrate stronger aversion to unfair proposals than fair ones, evidenced by higher rejection rates in UG; (2) Participants will exhibit greater cooperation (higher PGG contribution rates) with fair proposers compared to unfair counterparts; (3) Unfair proposals will elicit more negative MFN amplitudes, whereas fair proposals will evoke more positive P300 responses; (4) Neural activity (MFN/P300 amplitudes) triggered by unfairness experiences will significantly correlate with cooperative behavior in repeated games. By combining ERPs with sequential game paradigms, we bridge neurocognitive mechanisms and behavioral outcomes to decode how unfairness reprograms social decision-making.

## 3 Materials and methods

### 3.1 Participants

A priori power analysis was conducted using G^*^Power 3.1.9.7 (Faul et al., 2009). Following methodological recommendations for ERP studies (Hu and Mai, 2021; Wang et al., 2022), we set parameters with α = 0.05, power (1-β) = 0.80, and a medium effect size (*f* = 0.25). The analysis assumed default values for within-subject measurement correlation (ρ = 0.5) and non-sphericity correction (ε = 1.0), yielding a minimum required sample size of 28 participants. This power threshold (80%) aligns with established standards in ERP research where medium effect sizes are typically expected (Larson and Carbine, 2017). We successfully recruited 26 healthy university students, approaching our target sample size of 28. Two participants were excluded due to excessive artifacts in the EEG recordings. The final sample (*N* = 24) provided 75% power to detect medium effects (*f* = 0.25), exceeding sensitivity thresholds of observed large effects (*f* > 0.40). Ultimately, 24 participants (13 males; age range 18–21 years, *M*_age_= 19.50 ± 1.06) met all inclusion criteria: right-handedness, normal or corrected-to-normal vision, no neurological or psychiatric history, and no prior experience with economic games. The study protocol was approved by the Academic Ethics Committee of Jinan University. Participants received ¥50–60 compensation after providing written informed consent.

### 3.2 Experimental design

**Figure 1:**
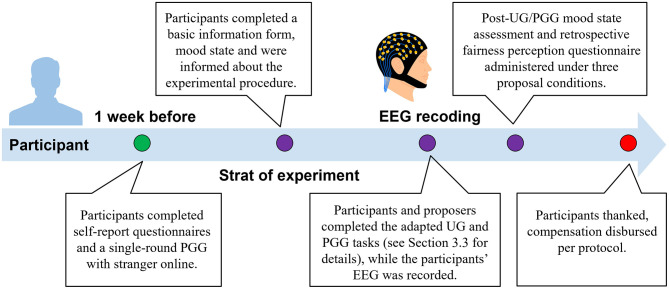
Overview of the experimental procedure.

The study employed a single-factor within-subjects experimental design. The independent variable was the type of proposal scheme, comprising three levels: fair, moderately unfair, and extremely unfair. Dependent variables included: the frequency of proposal rejection in the UG, evoked EEG components, and the amount of money contributed in the PGG. The complete experimental procedure is illustrated in Figure 1.

### 3.3 Task and procedure

This study established a dyadic interactive simulation paradigm. We first employed the UG to manipulate participants' perceived unfairness (fair, moderately unfair, extremely unfair), followed by the PGG to measure cooperation intentions during social interactions, thereby testing how unfairness experiences predict cooperative behavior. Critically, each trial involved a new anonymous partner pair. Participants were explicitly instructed: “*You will interact with different strangers every round—each UG proposer and PGG co-player is unique and will never reappear*.” This *one-shot stranger paradigm* eliminated reputation effects while allowing clean measurement of unfairness spillover.

#### 3.3.1 Ultimatum game

The UG, a well-established paradigm for studying fairness perception (Hu and Mai, 2021; Spape and Dundas, 2020), was implemented with participants explicitly assigned the role of Responder, while the Proposer was portrayed as an anonymous same-sex opponent. Critically, each proposer appeared only once in the entire experiment (one-shot design); participants were instructed: “Every offer comes from a new stranger you'll never see again.” Each trial began with a joint endowment of ¥10. Participants were informed that the Proposer would propose a monetary split, and they (as Responders) could either accept or reject it. Acceptance resulted in the proposed allocation, whereas rejection led to zero earnings for both parties. Critically, allocation schemes were preprogrammed to include three experimental conditions: fair (self/other = 5/5), moderately unfair (self/other = 3/7 or 4/6) and extremely unfair (self/other = 1/9 or 2/8). Control trials presented advantageous allocations (self/other = 9/1, 8/2, 7/3, 6/4) to counterbalance proposal valence and reduce predictability. These were excluded from analysis as we focused on disadvantageous inequity. Each experimental condition was presented 48 times (144 trials total), with 24 control trials, yielding 168 trials. Trail sequences were fully randomized across all conditions to mitigate order effects.

**Figure 2:**
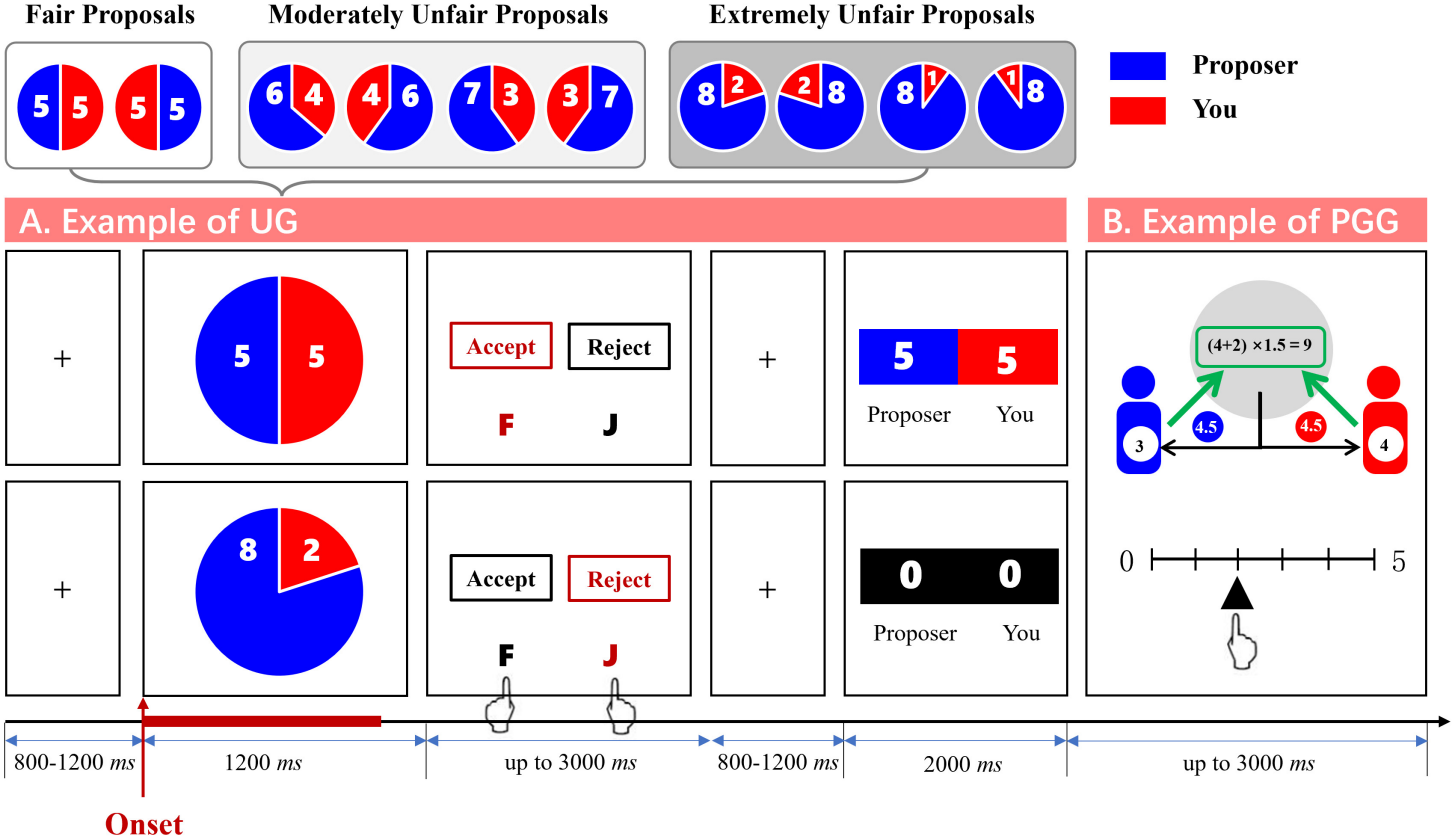
A single trial in the adapted Ultimatum Game **(A)** and Public Goods Game **(B)**.

A representative trial (e.g., self/other = 5/5 or 2/8; see Figure 2) proceeded as follows: each trial began with a fixation cross (+) appeared centrally for 800–1,200 ms. The allocation scheme (self in red, other in blue) was displayed for 1,200 ms. Participants responded within 3,000 ms (F = accept, J = reject). Unresponsive trials triggered a timeout warning and default rejection; Feedback (2,000 ms) showed the outcome: accepted proposals displayed the original split; rejected proposals showed zero earnings. Participants proceeded to the post-UG investment task (Section 3.3.2).

#### 3.3.2 Public goods game

The PGG, a validated measure of cooperation in social dilemmas (Dou et al., 2018; Parks et al., 2013). Adapting a validated two-phase approach to assess cooperative behavior, we measured: (a) Baseline cooperation (Phase 1): 1 week before the lab session, all participants completed an online single-round PGG after filling demographic survey. Upon receiving a ¥5 bonus via WeChat transfer, they were instructed: “You and an anonymous co-player (Player A) will simultaneously contribute 0–5 yuan to a public pool by scanning the experimenter's payment QR code. The pooled amount will be multiplied by 1.5 and divided equally between both players.” To ensure task comprehension, participants solved two calculation questions (e.g., “If you contribute ¥2 and Player A contributes ¥4, your final income = ¥4.5?”). Only those answering both correctly proceeded. The contribution amount served as the baseline cooperation index (T1).

(b) Post-manipulation cooperation (Phase 2): following each UG trial, participants engaged in an adapted repeated PGG (rPGG) with the same anonymous proposer from the immediately preceding UG interaction (i.e., the partner who had just made the monetary offer). This paired interaction design directly tested behavioral spillover effects. Each round began with a ¥5 endowment for both players. Using F/J keys to adjust a slider, participants privately contributed 0–5 yuan to a public pool (no real-time feedback on co-player's choice). Unbeknownst to participants, the co-player's contribution was programmatically matched to their own. The pool was multiplied by 1.5 and shared equally. In this task, the mean contribution to the public pool across the three UG proposal conditions (fair, moderately unfair, extremely unfair) was operationalized as post-manipulation cooperative behavior (i.e., mean contribution). Furthermore, to control for individual differences in baseline cooperation tendencies, we computed a Δ Cooperation score by subtracting baseline cooperation levels (T1, measured in Phase 1) from post-manipulation cooperation levels (T2, measured in Phase 2).

### 3.4 Experimental control and manipulation check

To control for potential confounding variables, participants completed self-report measures assessing key extraneous factors, including gender (Irwin et al., 2015), mood state (Dou et al., 2018), dispositional trust (Bianchi and Brockner, 2012), and empathic concern (Kowalski et al., 2015). Sex was recorded using dummy coding (0 = female, 1 = male) to account for potential sex differences in unfairness experience and cooperative behavior. Dispositional trust was evaluated using a five-item questionnaire (Schuessler, 1982) on a seven-point Likert scale (1 = *strongly disagree*, 7 = *strongly agree*), with higher scores reflecting greater trust propensity. Empathic concern was assessed using a six-item subscale from the Interpersonal Reactivity Index (Davis, 1980), also on a seven-point Likert scale (1 = *strongly disagree*, 7 = *strongly agree*), with higher scores indicating greater empathic concern. Mood state was measured using the *Positive and Negative Affect Scale* (PANAS; Watson et al., 1988), which includes 10 items assessing positive and negative affect on a seven-point Likert scale (1 = *not at all*, 7 = *extremely*), with higher scores indicating more pronounced mood states.

Specifically, participants first reported their mood states before completing the initial task. After completing the UG and PGG tasks, they required to complete PANAS and a retrospective fairness perception questionnaire under different proposal condition on a seven-point Likert scale (1 = *very unfair*, 7 = *very fair*), with higher scores indicating a stronger perception of fairness. Participants were instructed to reflect on their real-time experiences and the opponent's behavior during the task to provide accurate ratings. This approach ensured that both their immediate emotional state and their retrospective fairness judgments were accounted for in the analysis.

### 3.5 EEG recording and data analysis

Electroencephalogram (EEG) data were continuously recorded using a 64-channel BrainAmp Standard system (Brain Products, Munich, Germany) at a sampling rate of 500 Hz. Electrodes were positioned according to the 10–10 International system using Ag/AgCl ring electrodes. Vertical electrooculogram (VEOG) was monitored via electrodes placed 1 cm above and below the left eye, and horizontal EOG (HEOG) via electrodes at the outer canthi of both eyes. During recording, FCz (standard in 10–10 system) served as reference and AFz as ground, with all impedances maintained below 10 kΩ.

Offline preprocessing in BrainVision Analyzer 2 (Brain Products) followed ARTEMIS standards and (Keil et al. 2014) guidelines. Re-referencing to averaged mastoids (TP9/TP10), followed by ICA-based ocular artifact correction (Jung et al., 2000). The signal was filtered by a 0.05–30 Hz digital band-pass filter (24 dB/octave). The EEGs were segmented from 200 ms before to 800 ms after the presentation of the offer, and baseline correction was performed based on the 200 ms interval before the offer was presented. Segments containing maximum amplitudes that exceeded an absolute value of 100 μV or a voltage step from one sample to the next of 50 μV were excluded by means of automated artifact rejection. Finally, the EEG epochs were averaged separately for the fair, moderately unfair, and extremely unfair. MFN and P300 components were quantified based on grand-averaged waveforms and topographic maps across 24 participants. Temporal windows were defined as: MFN: 260–400 ms (negative peak amplitude within window; Miraghaie et al., 2022); P300: 450–650 ms (positive peak amplitude; Xu et al., 2020). Statistical analyses employed repeated-measures ANOVA. Greenhouse-Geisser correction addressed sphericity violations, with Bonferroni adjustment for multiple comparisons. All conditions retained >40 artifact-free trials after averaging.

## 4 Results

### 4.1 Manipulation check results

We conducted a two-way 3 (proposal scheme) and 2 (gender) repeated measure ANOVA for perceived fairness. There was a significant main effect of proposal scheme, *F*_(2, 44)_ = 60.35, *p* < 0.001, $\eta_{\text{p}}^{2}$ = 0.73. *Post-hoc* comparisons confirmed that the perceived fairness of *fair proposal* (self/other = 5:5) were significantly than *moderately unfair* (4:6 or 3:7) and *extremely unfair* proposals (2:8 or 1:9) (*ps* < 0.001). However, the main effect of gender [*F*_(1, 22)_ = 0.57, *p* = 0.46, $\eta_{\text{p}}^{2}$ = 0.03] and the interaction effect of these two factors were not significant [*F*_(2, 44)_ = 1.68, *p* = 0.21, $\eta_{\text{p}}^{2}$ = 0.07]. These results confirm the validity of the UG manipulation in eliciting graded unfairness perceptions and indicate that gender differences did not confound the experimental effects.

### 4.2 Covariables check results

The correlation analysis revealed significant negative correlations between dispositional trust and cooperative behavior under both moderately unfairness (*r* = −0.50, *p* < 0.05) and extremely unfairness conditions (*r* = −0.53, *p* < 0.05). Additionally, empathic concern was negatively correlated with cooperative behavior in the moderately unfairness condition (*r* = −0.41, *p* < 0.05). However, no significant correlations were found between other covariates and rejection rates in the UG or contribution in the PGG. Critically, supplementary analyses showed no significant associations between any covariates and MFN amplitudes (*ps* > 0.05) and P300 amplitudes (*ps* > 0.05). These results suggest that dispositional trust and empathic concern may influence inequity aversion or cooperation during the interaction, and thus should be controlled as covariates in subsequent analyses.

### 4.3 Behavioral results

#### 4.3.1 Rejection rates and response times in the UG

**Figure 3:**
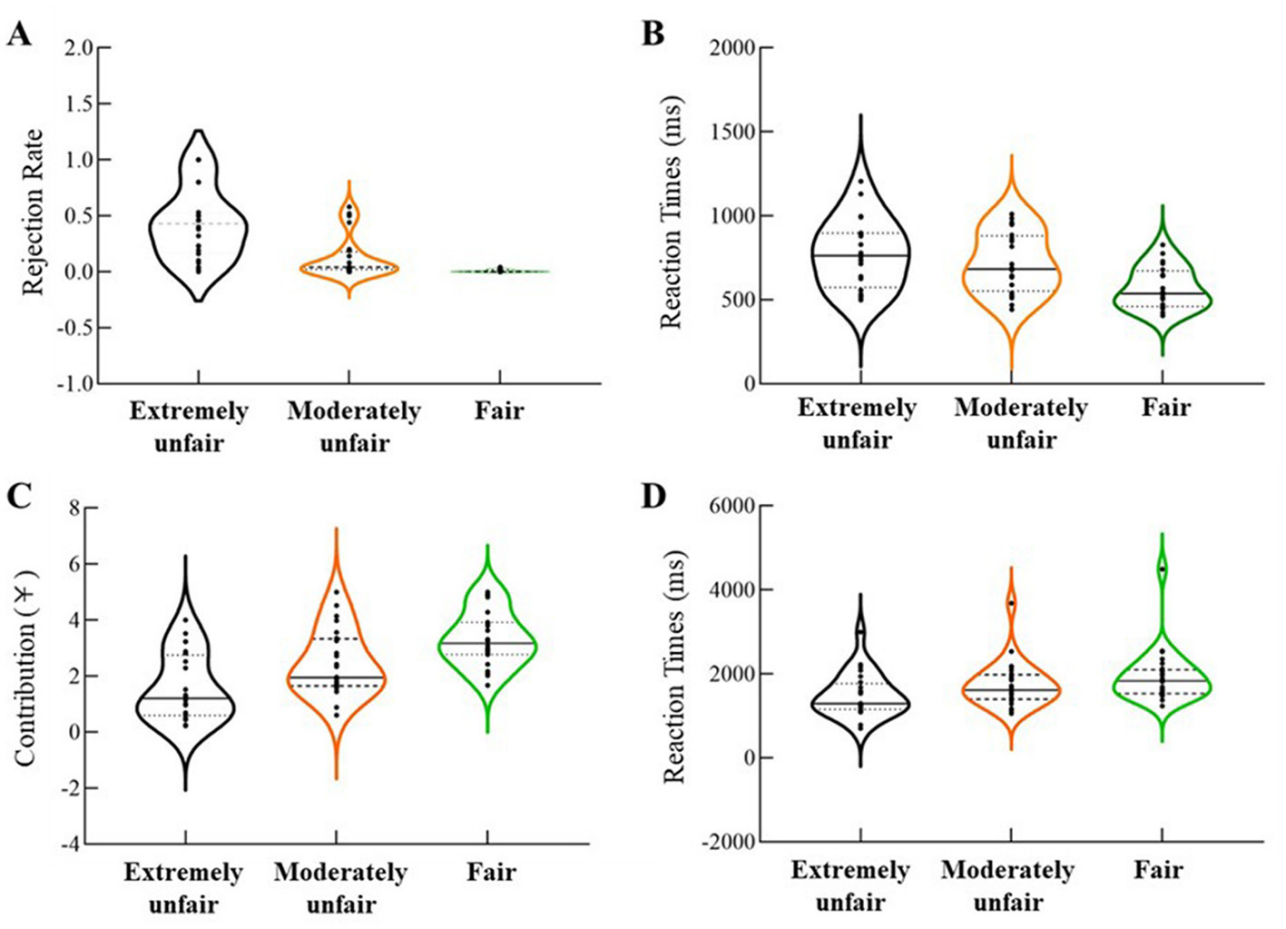
Behavioral results. **(A)** Rejection rates across three proposal conditions in the UG. **(B)** Response times for decision-making under three proposal conditions in the UG. **(C)** Manipulation-level contribution in the PGG across three UG proposal conditions. **(D)** Response times in PGG investment decisions three UG proposal conditions.

Repeated-measures ANOVAs were conducted with type of proposal scheme as the independent variable and rejection rates/response times (RTs) in the UG as dependent variables. For the rejection rates, as illustrated in Figure 3A, there was a significant main effect of proposal scheme, *F*_(2, 46)_ = 42.51, *p* < 0.001, η_p_^2^ = 0.65. *Post-hoc* analyses revealed that rejection rates for extremely unfair offers (*M* = 0.42 ± 0.06, *p* < 0.001) and moderately unfair offers (*M* = 0.13 ± 0.04, *p* = 0.003) were significantly higher than those for fair offers (*M* = 0.01 ± 0.01). Additionally, rejection rates for extremely unfair offers were significantly higher than those for moderately unfair offers (*p* < 0.001). For the RTs, as shown in Figure 3B, the main effect of proposal scheme was also significant, *F*_(2, 46)_ = 45.53, *p* < 0.001, η_p_^2^ = 0.66. *Post-hoc* analyses indicated that RTs for fair offers (*M* = 568.49 ± 24.50 ms) were significantly faster than those for moderately unfair (*M* = 717.40 ± 36.32 ms, *p* < 0.001) and extremely unfair offers (*M* = 774.91 ± 41.24 ms, *p* < 0.001). Furthermore, RTs for moderately unfair offers were significantly faster than those for extremely unfair offers (*p* = 0.03).

#### 4.3.2 Cooperative behavior and RT in the PGG

Repeated-measures ANOVAs were performed with the type of proposal scheme as the independent variable and contribution amounts/RTs in the PGG as dependent variables. For the contribution, as illustrated in Figure 3C, there was a significant main effect of proposal scheme, *F*_(2, 46)_ = 51.29, *p* < 0.001, η_p_^2^ = 0.69. *Post-hoc* analyses showed that contribution amounts after moderately unfair (*M* = 2.44 ± 0.24, *p* < 0.001) and extremely unfair offers (*M* = 1.57 ± 0.24, *p* = 0.002) were significantly lower than those under fair conditions (*M* = 3.32 ± 0.20). Contributions under extremely unfair conditions were also significantly lower than moderately unfair conditions (*p* < 0.001).

For the RTs in investment decisions, as illustrated in Figure 3D, the main effect of proposal scheme was significant, *F*_(2, 46)_ = 26.46, *p* < 0.001, η_p_^2^ = 0.54. *Post-hoc* analyses found that RTs for decisions involving fair proposers (*M* = 1940.05 ± 132.69 ms) were significantly slower than those for moderately unfair (*M* = 1749.67 ± 111.92 ms, *p* = 0.01) and extremely unfair proposers (*M* = 1489.87 ± 104.87 ms, *p* < 0.001). RTs for moderately unfair proposers were also significantly slower than those for extremely unfair proposers (*p* < 0.001).

### 4.4 ERP results

#### 4.4.1 MFN (260–400 ms)

**Figure 4:**
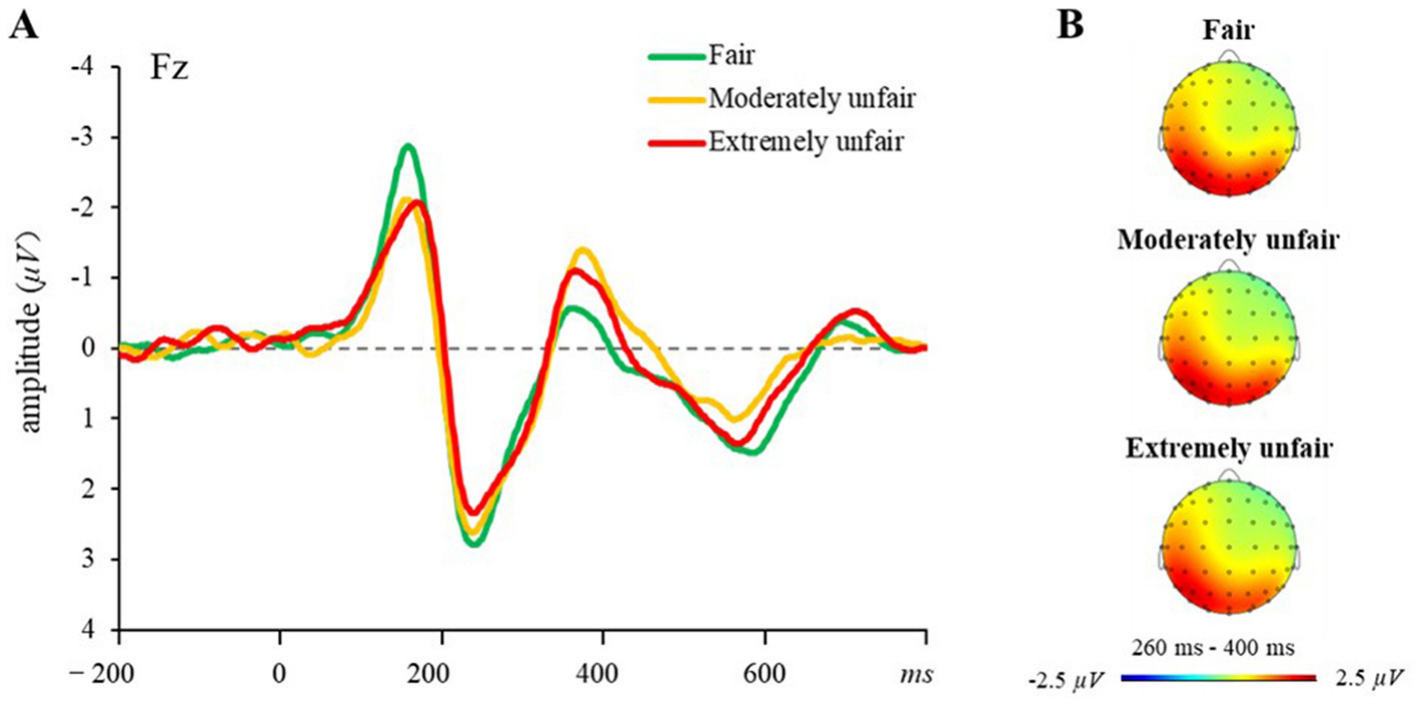
MFN results. **(A)** Grand-averaged waveforms at electrode Fz across three proposal condition in the UG. **(B)** Topographic maps for the MFN in the 260–400 ms time window.

Figure 4 illustrates the grand-averaged ERP waveforms at Fz and the topographies of MFN in three proposal conditions over the 260–400 ms time interval. We conducted a two-way 3 (proposal scheme: fair, moderately unfair, extremely unfair) × 3 (electrode site: Fz, FCz, Cz) repeated measure ANOVA for peak amplitudes of MFN. There were no significant main effect for proposal scheme [*F*_(2, 46)_ = 1.69, *p* = 0.19, $\eta_{\text{p}}^{2}$ = 0.07] and electrode site [*F*_(2, 46)_ = 1.95, *p* = 0.15, $\eta_{\text{p}}^{2}$ = 0.08]. However, the interaction between proposal scheme and electrode site was significant, *F*_(4, 92)_ = 2.61, *p* < 0.05, $\eta_{\text{p}}^{2}$ = 0.10. Simple effects analysis indicated that at the Fz electrode, moderately unfair offers (*M* = −2.35 μV, *SE* = 0.31) and extremely unfair offers (*M* = −2.21 μV, *SE* = 0.25) elicited significantly more negative MFN amplitudes compared to fair offers (*M* = −1.74 μV, *SE* = 0.26; all *ps* < 0.05). No significant differences were observed across proposal conditions at other electrode sites (*ps* > 0.05).

#### 4.4.2 P300 (450–650 ms)

**Figure 5:**
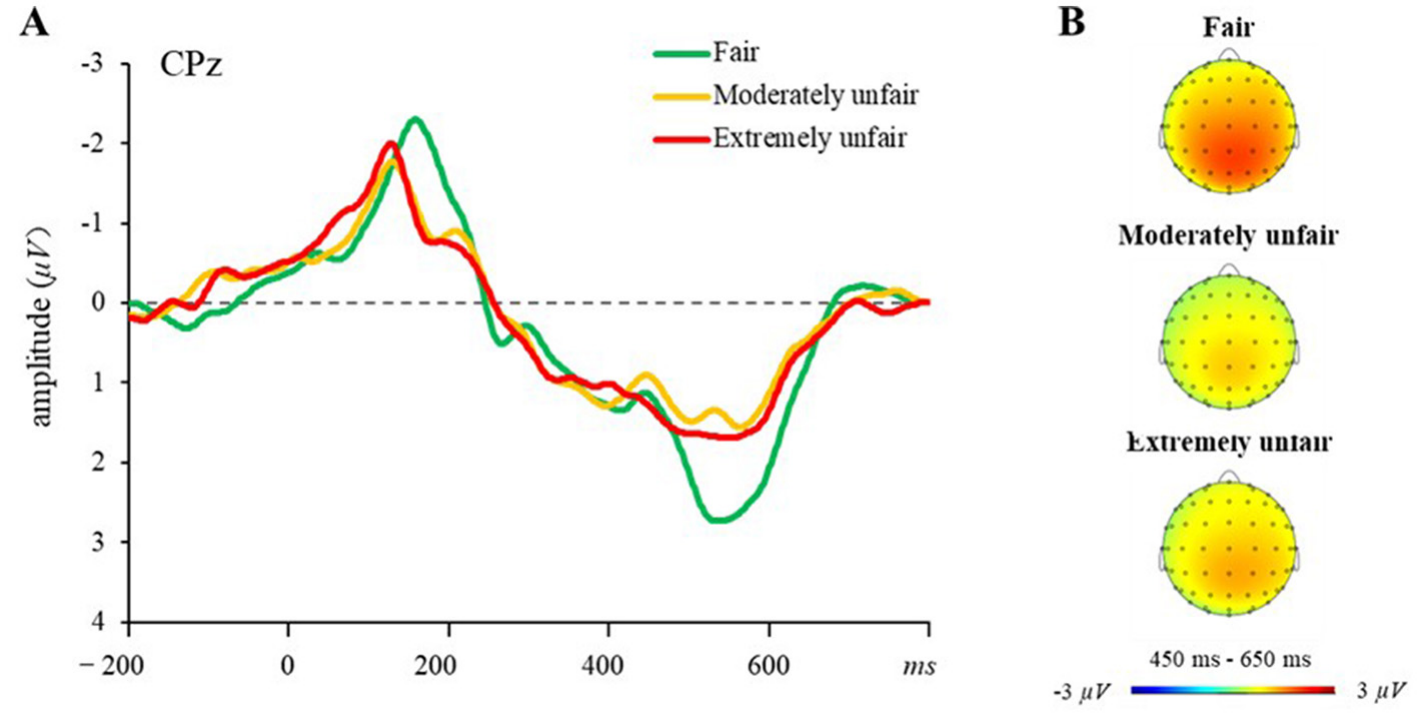
P300 results. **(A)** Grand-averaged waveforms at electrode CPz across three proposal conditions in the UG. **(B)** Topographic maps for the P300 in the 450–650 ms time window.

Figure 5 illustrates the grand-averaged ERP waveforms at CPz and the topographies of P300 in three proposal conditions over the 450–650 ms time interval. We conducted a two-way 3 (proposal scheme: fair, moderately unfair, extremely unfair) × 5 (electrode site: Fz, FCz, Cz, CPz, Pz) repeated measure ANOVA for peak amplitudes of P300. A significant main effect of proposal scheme was observed, *F*_(2, 46)_ = 4.65, *p* < 0.05, $\eta_{\text{p}}^{2}$ = 0.17). *Post-hoc* analyses showed that the fair offers elicited significantly larger P300 (*M* = 3.24 μV, *SE* = 0.329) compared to moderately unfair offers (*M* = 2.50 μV, *SE* = 0.24). However, no significant difference was found between moderately unfair offers and extremely unfair offers. The main effect of electrode site was also significant, *F*_(4, 92)_ = 6.47, *p* < 0.001, η_p_^2^ = 0.22. *Post-hoc* analyses demonstrated hierarchical amplitude reductions: P300 amplitudes at Fz were significantly smaller than those at FCz, Cz, CPz, and Pz (*ps* < 0.05); amplitudes at FCz were smaller than those at Cz and CPz (*ps* < 0.01); and amplitudes at Cz were smaller than those at CPz (*p* < 0.01). The interaction between proposal scheme and electrode site was significant, *F*_(8, 184)_ = 2.15, *p* < 0.05, $\eta_{\text{p}}^{2}$ = 0.09. Simple-effects analysis of the interaction effect revealed that fair offers elicited larger P300 amplitudes than moderately unfair offers at Fz, FCz, and Cz. Fair offers elicited larger P300 amplitudes compared to moderately unfair and extremely unfair offers at CPz and Pz.

### 4.5 Results of neural-behavioral correlation

To test our primary hypothesis regarding how generalized unfairness processing (vs. fairness) shapes cooperation, we employed a binary classification approach within a neural-behavioral integration framework. This consolidation serves three key purposes: (a) maximizes statistical power for mediation analyses, (b) reduces model complexity given our focus on broad unfairness effects, and (c) aligns with established methods examining global inequity aversion (e.g., Sanfey et al., 2003). Unfair experiences were dummy-coded (0 = fair, 1 = moderately or extremely unfair) and correlated with cooperation (T2), Δ Cooperation (T2–T1), UG rejection rates, amplitudes of MFN and P300.

**Figure 6:**
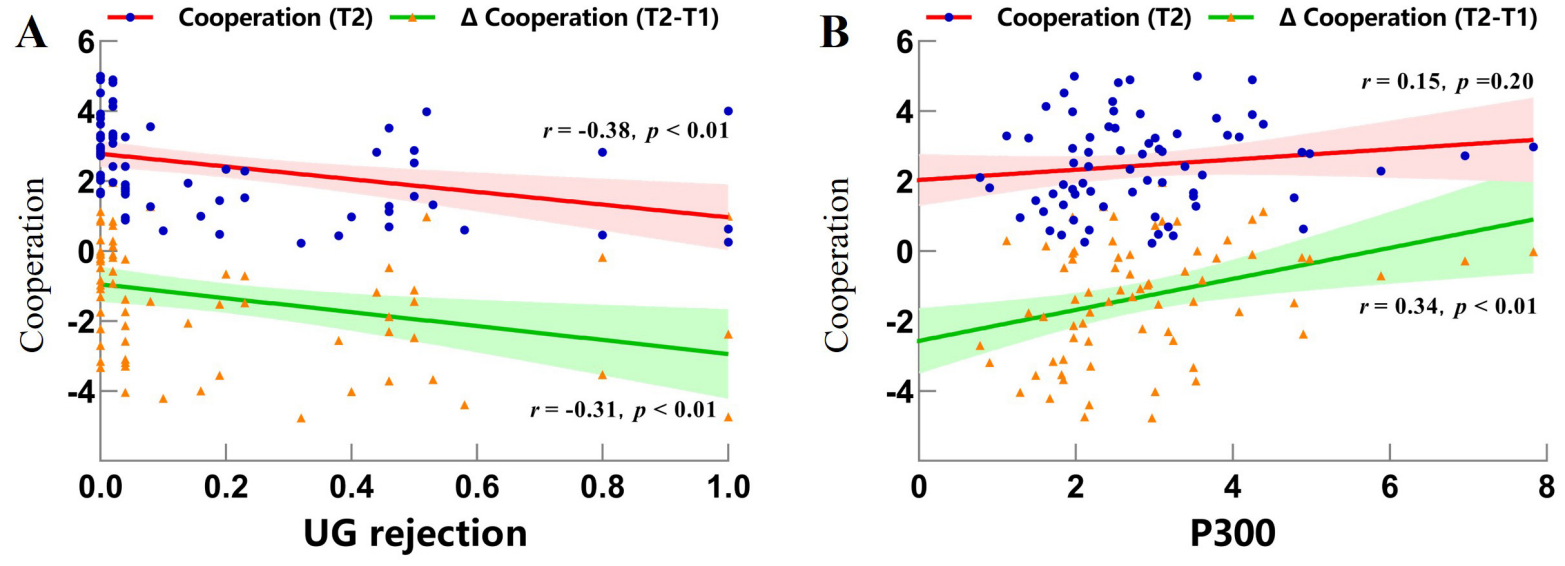
Scatterplots of key neural-behavioral correlations. **(A)** Correlation between UG rejection rates and cooperation; **(B)** Correlation between P300 amplitudes and cooperation.

Results revealed significant negative correlations between unfair experiences and both T2 cooperation (*r* = −0.48, *p* < 0.001) and Δ Cooperation (*r* = −0.36, *p* = 0.004). As illustrated in Figure 6A, UG rejection rates were also negatively correlated with T2 cooperation (*r* = −0.38, *p* = 0.002) and Δ Cooperation (*r* = −0.31, *p* = 0.009). Notably, as illustrated in Figure 6B, P300 amplitudes showed a significant positive correlation with Δ Cooperation (*r* = 0.34, *p* = 0.006), while MFN amplitudes exhibited no significant correlations with any behavioral measures (*ps* > 0.05). These findings indicate that participants exhibiting reduced P300 amplitudes during unfair (vs. fair) conditions showed diminished adaptive adjustments in cooperation. This pattern of correlations is consistent with a potential mediating role of P300 in the neurobehavioral pathway linking unfairness to cooperation, though formal mediation testing requires future investigation.

## 5 Discussion

Fairness serves as a critical social norm governing interpersonal interactions and societal stability (Li et al., 2025; Lucca et al., 2018). This study employed ERPs and adapted paradigms—the UG and PGG—to investigate the dynamic impact of unfair experiences on cooperative behavior and their underlying neural mechanisms. Our findings elucidate behavioral patterns of inequity aversion in interactive games and their neurophysiological correlates, advancing the understanding of how fairness preferences regulate cooperative decision-making.

Consistent with Hypothesis 1, participants exhibited significantly higher rejection rates for moderately and extremely unfair offers compared to fair ones in the UG task, demonstrating robust inequity aversion. This aligns with Fehr and Schmidt's (1999) *inequity aversion model*, which posits that individuals evaluate both absolute gains and relative disparities in resource allocation. Our results corroborate prior evidence that people prefer fair distributions and reject unfair ones (Fan et al., 2024; Fernandes et al., 2019), particularly under disadvantageous inequity (Bechtel et al., 2018; Fan et al., 2024; Li et al., 2025; Yu et al., 2014). These findings challenge the *homo economicus* assumption by highlighting that fairness concerns—not just self-interest—drive decision-making in social dilemmas. Critically, exposure to disadvantageous inequity suppressed cooperative behavior in subsequent PGG trials, corroborating Hypothesis 2. This extends the scope of inequity aversion as a social preference (Côté et al., 2015; Nishi et al., 2015).

Specifically, unfair experiences may activate self-protective motives (e.g., fear of exploitation) or negative emotions (e.g., anger), prompting individuals to punish unfair proposers through non-cooperation (Güroglu et al., 2010; Rilling and Sanfey, 2011). Notably, the decision dynamics in the UG phase revealed a distinctive RT pattern: responses to moderately unfair offers were significantly faster than those to extremely unfair offers, diverging from classic standalone UG studies where moderately unfair offers typically elicit the longest RTs (e.g., Fabre et al., 2015; Nicolaisen-Sobesky et al., 2023; Polezzi et al., 2008). We propose this stems from the chained paradigm's motivational shift: participants evaluated UG offers not merely as isolated fairness decisions, but as signals of the co-player's social intent for imminent PGG cooperation. Moderately unfair offers may be interpreted as ambiguous but tolerable violations, enabling efficient “accept-to-cooperate” decisions. Conversely, extremely unfair offers signaled exploitative intent, triggering prolonged conflict between punishment motives and cooperative goals. This interpretation is reinforced by PGG RTs: rapid decisions after extreme unfairness reflect emotion-driven disengagement, while slower deliberation after fairness aligns with strategic cooperation optimization. Such retaliatory behaviors reflect a prioritization of fairness over material gains in cooperative contexts.

ERPs results revealed distinct neural signatures of fairness processing. Both moderately and extremely unfair proposals elicited enhanced MFN compared to fair offers, indicating heightened attentional resource allocation toward norm-deviant outcomes. This aligns with Hypothesis 3 and corroborates prior UG studies demonstrating that disadvantageous inequity amplifies MFN amplitudes (Hu and Mai, 2021; Kessler et al., 2017; Wang et al., 2017). The MFN reflects the discrepancy between actual outcomes and socially anchored predictions (Falco et al., 2019; Spape and Dundas, 2020), suggesting that unfair proposals violate deeply ingrained equity norms. As a neural marker of expectancy violation (Falco et al., 2019; Spape and Dundas, 2020), the amplified MFN reflects the perceived deviation of unfair proposals from deeply internalized fairness norms. Notably, the absence of significant MFN differences between moderate and extreme inequity may stem from the heightened cognitive ambiguity of moderately unfair offers, which require greater mentalizing effort (Wang et al., 2017).

Conversely, fair proposals evoked larger P300 amplitudes—a component linked to the integration of emotional salience and attentional prioritization (Hu and Mai, 2021; Leng and Zhou, 2010; Li et al., 2025; Sanfey et al., 2003). The enhanced P300 to fair outcomes aligns with their role as intrinsic rewards that align with social expectations (Bellebaum et al., 2010; Kobza et al., 2011), reinforcing the motivational value of norm-congruent outcomes. Exploratory neurobehavioral analyses partially supported Hypothesis 4: P300 amplitudes during UG trials positively related to subsequent cooperation levels in the PG, whereas MFN responses showed no significant association. This tentatively positions reward valuation (indexed by P300), rather than conflict detection (indexed by MFN), as a neural precursor of post-inequity cooperation. While preliminary, these findings offer novel insights into how neural reward systems may scaffold cooperative recovery following unfair experiences.

These findings hold significant theoretical and practical implications. Theoretically, they elucidate the competitive interplay between fairness preferences and loss aversion in interactive games, advancing models of social decision-making that account for dual motivational systems. Practically, the identified neural and behavioral mechanisms offer actionable insights for fostering cooperation in real-world settings. We propose two concrete evidence-based organizational strategies: proactive mitigation of inequality impacts through institutional safeguards in compensation systems and early-intervention protocols for high-unfairness-perception employees, directly countering observed cooperation suppression; and fairness-preference-integrated collaboration frameworks requiring formalized equity norms in resource allocation policies alongside cooperation-contingent incentive structures that reinforce fairness-contribution linkages. However, several limitations warrant consideration. First, methodological constraints require attention: the experimental design—while rigorously controlled—entails two interpretative caveats. Specifically, the use of confederates potentially attenuates ecological validity in social dynamics, and non-counterbalanced response-hand assignments in the UG may introduce motor artifacts, despite mitigation through: full trial randomization dispersing motor effects, and rigorous preprocessing (including ocular artifact removal and motor-related component exclusion). Second, the homogeneous sample—drawn exclusively from university students—limits the generalizability of findings to broader populations with diverse socioeconomic backgrounds. Third, while neuro-behavioral correlations suggest potential pathways (e.g., P300 → Δ Cooperation), formal mediation analyses were not conducted; future research should employ path modeling to verify these neurocognitive mechanisms. Fourth, computational modeling could formalize dynamic fairness-cooperation relationships in naturalistic, multi-round interactions. Fifth, although our final sample size of 24 participants provided 75% power to detect medium effects (*f* = 0.25), it falls short of the optimal power level of 80% recommended in ERP studies. This limitation may affect the robustness of our findings and should be addressed in future research with larger sample sizes.

## 6 Conclusions

This study demonstrates that unfair experiences reduce cooperation through neurocognitive mechanisms. Participants exhibited inequity aversion, rejecting unfair proposals and withholding cooperation, prioritizing fairness over self-interest. Neurophysiologically, unfair proposals heightened MFN (norm violation detection), while fair outcomes amplified P300 (reward processing), with P300 positively related to subsequent cooperation. These neurobehavioral findings robustly support the *inequity aversion* effect, highlighting the neural mechanisms through which fairness norms and reward valuation jointly govern cooperative behavior.

## Data Availability

The raw data supporting the conclusions of this article will be made available by the authors, without undue reservation.
